# Endometriosis-Related Impairment in Assisted Reproductive Technologies: Inflammatory Profiles, Oocyte Competence, and Embryo Development

**DOI:** 10.3390/jcm15051723

**Published:** 2026-02-25

**Authors:** Francesca Papini, Susanna Cappellini, Ilaria Marcacci, Ilaria Marzi, Elena Casarosa, Simona Daniele, Sara Macaluso, Amerigo Ferrari, Andrea Panattoni, Paolo Giovanni Artini, Vito Cela

**Affiliations:** 1 Division of Gynecology and Obstetrics, Department of Clinical and Experimental Medicine, University of Pisa, 56126 Pisa, Italy; francesca.papini@ao-pisa.toscana.it (F.P.); saramacaluso95@gmail.com (S.M.); a.ferrari27@studenti.unipi.it (A.F.);; 2 Department of Surgical Pathology, Medical Molecular and Critical Area, Institute of Legal Medicine, University of Pisa, 56126 Pisa, Italy; 3 Department of Pharmacy, University of Pisa, 56126 Pisa, Italy

**Keywords:** endometriosis, ART, cytokines

## Abstract

**Background:** Endometriosis is associated with infertility and impaired assisted reproductive technology (ART) outcomes, potentially due to an altered follicular microenvironment characterized by chronic inflammation. This study investigates the systemic and local inflammatory profiles in women with endometriosis and assesses their impact on oocyte and embryo quality using both static and dynamic embryo evaluation. **Methods:** A prospective, monocentric observational study enrolled 47 women undergoing controlled ovarian stimulation for ART, including 29 with laparoscopically confirmed endometriosis and 18 controls with tubal or male-factor infertility. Serum and follicular fluid cytokines (TGF-β1, NF-κB, IL-10, HIF-1α) were quantified. A sub-study analyzed embryo quality and development in 36 patients subdivided into static morphological assessment and dynamic time-lapse monitoring cohorts. **Results:** Endometriosis patients exhibited significantly elevated pro-inflammatory cytokines (TGF-β1, NF-κB) and reduced anti-inflammatory IL-10 in serum, alongside decreased NF-κB in follicular fluid. These alterations correlated with diminished ovarian reserve, reduced oocyte yield, and lower fertilization rates. Embryos from endometriosis patients showed increased multinucleation and persistent fragmentation, features more sensitively detected via dynamic time-lapse imaging. Clinical pregnancy rates were significantly lower in the endometriosis group. **Conclusions:** Endometriosis induces a dysregulated inflammatory follicular milieu that adversely affects oocyte competence and embryo morphodynamics. Dynamic embryo assessment provides enhanced detection of subtle developmental abnormalities. Integration of immunomodulatory strategies and advanced embryo monitoring may improve ART success in this population.

## 1. Introduction

Endometriosis is a chronic gynecological disorder affecting approximately 10% of women of reproductive age and is frequently associated with infertility [1]. The mechanisms through which endometriosis impairs reproductive outcomes are multifactorial and remain partially understood. Anatomical distortion due to pelvic adhesions and ovarian endometriomas has long been considered a primary factor. However, numerous studies suggest that the pathophysiological impact of endometriosis extends beyond structural alterations, involving chronic inflammation and oxidative stress within the follicular microenvironment [2,3,4]. A recent study has highlighted that follicular fluid from women with endometriosis exhibits elevated levels of inflammatory cytokines, contributing to a pro-inflammatory microenvironment that may impair oocyte quality [5].

In women undergoing assisted reproductive technology (ART), endometriosis has been associated with reduced fertilization rates, compromised embryo quality, and lower implantation and pregnancy rates compared to other infertility etiologies [6]. These clinical observations have prompted growing interest in investigating the local biochemical and cellular environments of the ovarian follicle. Follicular fluid, which surrounds the oocyte and reflects the metabolic and immunologic status of the follicle, has been found to contain elevated levels of inflammatory cytokines and reactive oxygen species in women with endometriosis [7,8,9].

This study was designed to explore these mechanisms using a two-tiered approach. The main study evaluated systemic and follicular cytokine profiles and ART outcomes in a cohort of women undergoing controlled ovarian stimulation (COS), comparing those with endometriosis to women with tubal or male factor infertility. The sub-study focused on identifying significant differences in oocyte and embryo quality and assessing the effectiveness of two evaluation systems—static and dynamic—in selecting the best quality embryos.

## 2. Methods

### 2.1. Study Design and Population

This prospective, monocentric observational study evaluated clinical and biological outcomes in women undergoing controlled ovarian stimulation (COS) for assisted reproductive technology (ART), comparing 29 patients with laparoscopically confirmed endometriosis to 18 control women with infertility due to tubal or male factor. All ART cycles were conducted at the Medically Assisted Reproduction Center, Department of Clinical and Experimental Medicine, University of Pisa, between October 2019 and March 2021.

All participants underwent a gonadotropin-releasing hormone (GnRH) antagonist protocol initiated in the early follicular phase (day 2–3 of the menstrual cycle). Initial gonadotropin dose and type were determined based on age, body mass index (BMI), and ovarian reserve, assessed by anti-Müllerian hormone (AMH) and antral follicle count (AFC). Ovarian response was monitored via transvaginal ultrasound and serum levels of estradiol and progesterone. A GnRH antagonist (0.25 mg/day) was introduced when at least one follicle reached a mean diameter of ≥14 mm. Final oocyte maturation was triggered with either recombinant human chorionic gonadotropin (rhCG, 250 µg) or a GnRH agonist (triptorelin 0.2 mg), depending on the risk of ovarian hyperstimulation syndrome (OHSS) or the embryo transfer strategy (fresh vs. frozen). Additional methodological details concerning the stimulation protocol and monitoring of the ovarian response was reported in Supplementary File S1.

Oocyte retrieval was performed 36 h post-trigger. Oocytes were inseminated via in vitro fertilization (IVF) or intracytoplasmic sperm injection (ICSI), based on semen quality. Embryo transfer (ET) occurred on day 2–5 post-fertilization (fresh or frozen). Serum beta-human chorionic gonadotropin (β-hCG) was measured 14 days after ET; values > 5 mUI/mL were considered positive.

Clinical parameters evaluated included age, BMI, baseline follicle-stimulating hormone (FSH), luteinizing hormone (LH), and estradiol (measured on day 3), AMH, AFC, total gonadotropin dose, stimulation duration, number of follicles ≥ 16 mm at trigger, and biochemical pregnancy rate. Biological outcomes included the number of retrieved oocytes, metaphase II (MII) oocytes, fertilized oocytes, embryo quality, blastocyst formation, and β-hCG positivity.

Serum and follicular fluid samples were collected at the time of oocyte retrieval, along with cumulus cells, and stored at –80 °C. Inflammatory markers—including nuclear factor kappa B (NF-κB), hypoxia-inducible factor 1-alpha (HIF-1α), transforming growth factor beta 1 (TGF-β1), and interleukin-10 (IL-10)—were measured via Western blot or enzyme-linked immunosorbent assay (ELISA), and normalized to total protein concentration assessed by Bradford assay. Extended methodological details regarding sample processing, protein quantification, and cytokine assays are provided in the Supplementary File S1.

### 2.2. Sub-Study

This sub-analysis involved 36 women undergoing ART, selected from the main study cohort and evenly distributed into two embryo evaluation protocols: static and dynamic. Each subgroup included 9 women with endometriosis and 9 age-matched controls with tubal or male-factor infertility and good reproductive prognosis. All cycles were performed using conventional IVF and embryo transfer (FIVET). Inclusion in the sub-study was based solely on the patient’s consent to undergo additional embryo evaluation procedures.

The static evaluation involved standard morphological assessment of embryo quality on days 2 and 3 post-insemination. The dynamic group used time-lapse imaging with continuous embryo monitoring via the GERI incubator system, which enabled quantitative analysis of early developmental kinetics and fragmentation. Key outcomes included fertilization rate, oocyte maturity, embryo grading, blastocyst formation and quality, cleavage-stage fragmentation, timing of early mitotic events, and incidence of multinucleation. Clinical outcomes were also compared between groups. Extended methodological details regarding embryo culture conditions, imaging technology, time-lapse metrics, and grading criteria are available in the Supplementary File S2. Furthermore, oocyte and embryo static and dynamic morphological assessment and the scoring system for blastocysts [10,11] are reported in Supplementary Table S1 and Supplementary Table S2, respectively.

### 2.3. Statistical Analysis

Statistical analyses were performed using GraphPad Prism 7 (GraphPad Software Inc., San Diego, CA, USA). Continuous clinical, hormonal, and laboratory variables were reported as mean ± standard deviation (SD) and compared using unpaired Student’s t-tests for normally distributed data or Mann–Whitney U tests for non-normally distributed data. Categorical variables were expressed as counts and proportions and compared using the chi-squared test. Analyses were conducted at the patient level for clinical, hormonal, and cytokine outcomes. Embryo-related parameters were summarized within each study group. A *p*-value < 0.05 was considered statistically significant.

## 3. Results

### 3.1. Main Study

A total of 47 patients undergoing ART were enrolled in the main study, including 29 women with confirmed endometriosis and 18 controls with infertility of tubal or male factor origin. Baseline characteristics are shown in Table 1. Women with endometriosis were slightly older (36.86 ± 3.68 vs. 35.20 ± 3.30 years, nonsignificant (ns)) and had significantly lower ovarian reserve, as shown by AMH levels (2.29 ± 0.44 vs. 2.91 ± 0.29 ng/mL; *p* = 0.009) and AFC (7.76 ± 0.54 vs. 14.22 ± 0.93; *p* < 0.001). No significant differences were found in age; BMI; and FSH, LH or estradiol levels.

While total gonadotropin dose and stimulation duration were similar between groups, the gonadotropin/day ratio was significantly higher in the endometriosis group (350.1 ± 18.9 vs. 285.4 ± 10.9, *p* < 0.05). The number of follicles > 16 mm at trigger was significantly lower in the endometriosis group (3.59 ± 1.82 vs. 6.22 ± 4.01, *p* = 0.004). Women with endometriosis had significantly fewer oocytes retrieved (3.86 ± 0.36 vs. 6.00 ± 0.62, *p* = 0.001), fewer mature MII oocytes (3.36 ± 0.32 vs. 4.94 ± 0.50, *p* = 0.004), and fewer fertilized oocytes (2.89 ± 0.35 vs. 4.11 ± 0.58, *p* = 0.031). There was no significant difference in the rate of visible oocyte morphological abnormalities.

No statistically significant differences were found in embryo quality (grades I–III) or blastocyst formation. However, a nonsignificant trend toward more grade I embryos and fewer grade III embryos in the control group was observed. The rate of positive β-hCG was significantly lower in patients with endometriosis (40% vs. 68%, *p* = 0.043), as shown in Table 2.

**Figure 1:**
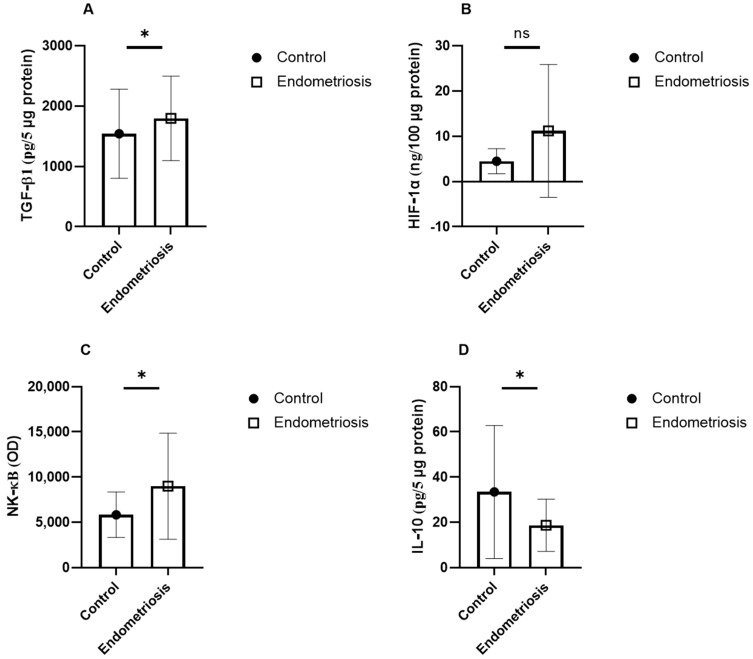
Serum protein levels of proinflammatory TGF-β1 (**A**), HIF-1α (**B**), NF-κB (**C**) and IL-10 (**D**) in samples from control patients affected by endometriosis. The data are reported as the mean values ± SD of three independent experiments, each performed in duplicate. Statistical analysis was performed by unpaired *t*-test: * *p* < 0.05 versus control, ns not significant.

**Figure 2:**
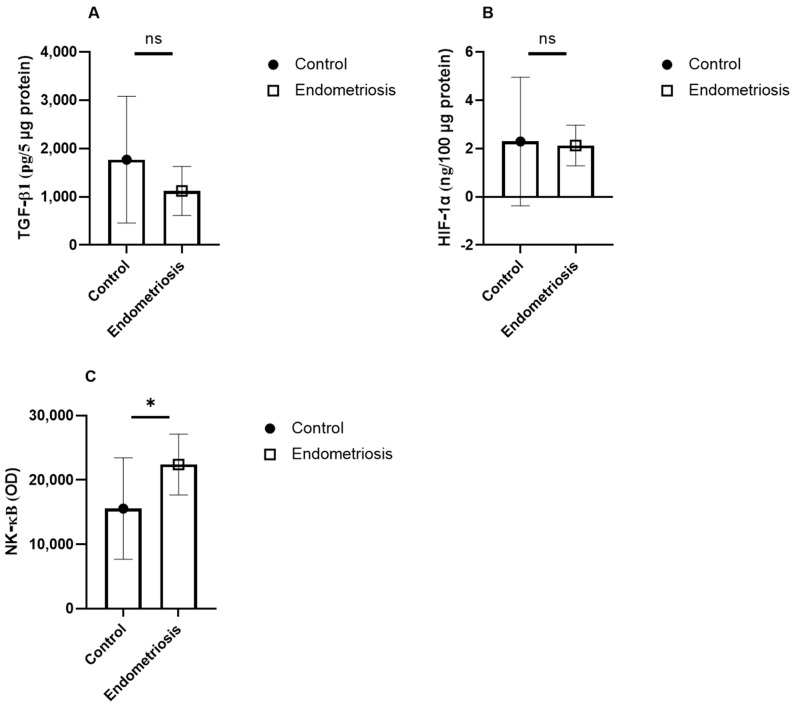
Follicolar Fluid (FF) protein levels of proinflammatory TGF-β1 (**A**), HIF-1α (**B**), NF-κB (**C**) in samples from control and patients affected by endometriosis. The data are reported as the mean values ± SD of three independent experiments, each performed in duplicate. Statistical analysis was performed by unpaired *t*-test: * *p* < 0.05 versus control, ns not significant.

In serum, patients with endometriosis showed significantly higher levels of TGF-β1 (1796 ± 699 vs. 1542 ± 738 pg/5 μg, *p* = 0.04) and NF-κB (8984 ± 5867 vs. 5827 ± 2510 RU, *p* = 0.03), and lower IL-10 (18.7 ± 11.5 vs. 33.4 ± 29.4 pg/5 μg, *p* = 0.04). HIF-1α levels were not significantly different. In follicular fluid, NF-κB levels were significantly higher in the endometriosis group (15,573 ± 7887 vs. 22,409 ± 47,231 RU, *p* = 0.04) (Table 3, Figure 1 and Figure 2). No significant differences were observed in TGF-β1 or HIF-1α concentrations.

Serum TGF-β1 positively correlated with FSH levels across the cohort. A non-significant trend was noted between follicular TGF-β1 and both estradiol at trigger and AMH (inverse correlation), as well as between serum HIF-1α and AMH (inverse) and FSH (direct).

### 3.2. Sub-Study

The sub-analysis included 36 patients from the main study cohort—18 women with endometriosis and 18 controls—subdivided equally between two embryo assessment protocols: static and dynamic. Patients with endometriosis were significantly older than controls (37.11 ± 2.87 vs. 34.36 ± 2.37 years, *p* = 0.005). The mean number of retrieved oocytes was significantly lower in the endometriosis group (6.56 ± 3.36 vs. 9.86 ± 3.37, *p* = 0.01), as was the oocyte retrieval rate per aspirated follicle (67% vs. 82%). The number of MII oocytes (5.61 ± 3.35 vs. 8.29 ± 3.63, *p* = 0.04), 2PN zygotes (3.78 ± 3.94 vs. 6.43 ± 3.08, *p* = 0.01), and developmentally competent embryos (2.67 ± 1.94 vs. 4.29 ± 2.05, *p* = 0.03) were also significantly reduced in women with endometriosis compared to controls. Embryo quality differed significantly between groups, with fewer grade I embryos in the endometriosis group (0.33 ± 0.59 vs. 1.86 ± 1.23, *p* = 0.0004) and a higher number of grade III embryos (0.78 ± 1.00 vs. 0.21 ± 0.43, *p* = 0.04).

No statistically significant differences were observed for the number of aspirated follicles, incidence of abnormal fertilization (1PN/3PN), unfertilized oocytes, MI or GV-stage oocytes, number of non-developing embryos, number of grade II embryos, total number of blastocysts formed, or their expansion stage.

**Figure 3:**
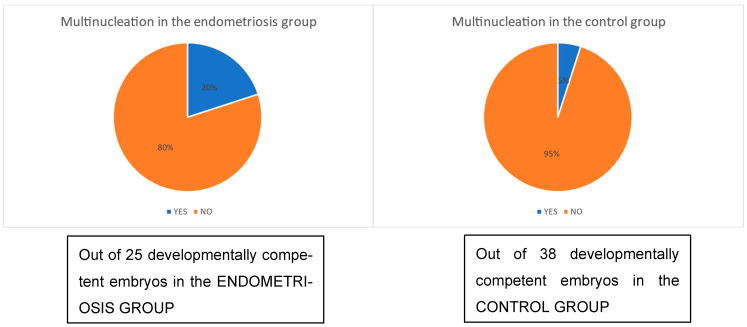
Comparison between the endometriosis group and the control group for the parameter “embryo multinucleation.” Dynamic method.

**Figure 4:**
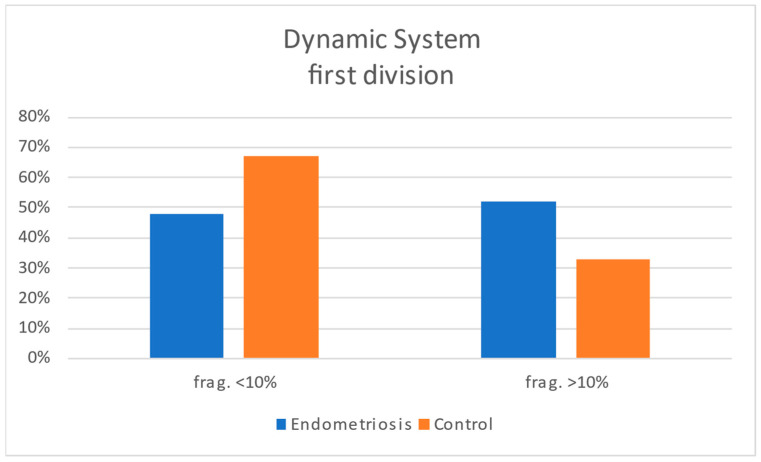
Histogram comparing the degrees of embryonic fragmentation between case and control groups at the first cell division in the dynamic study.

**Figure 5:**
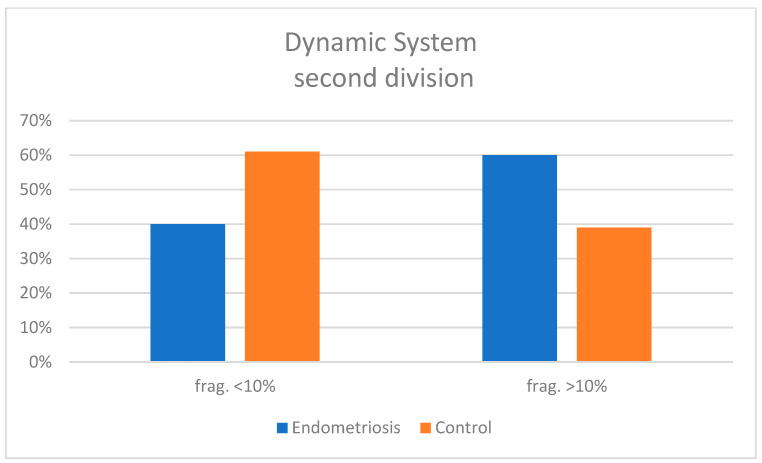
Histogram comparing the degrees of embryonic fragmentation between case and control groups at the second cell division in the dynamic study.

In the dynamic subgroup, the timing of early embryonic events—including 2PN fusion, first and second cell divisions, and duration of the two-cell stage—did not differ significantly between groups, whether analyzed across all 2PN zygotes or only among developmentally competent embryos (Table 4). However, multinucleation was observed more frequently in embryos from endometriosis patients (20%) compared to controls (5%) (Figure 3). Fragmentation analysis also showed a higher incidence of moderate-to-severe fragmentation in the endometriosis group at both the first (52% > 10% fragments vs. 33% in controls) (Figure 4) and second cell divisions (60% > 10% vs. 39%) (Figure 5). In contrast, embryos from controls showed a greater tendency to reduce or maintain initial fragmentation levels, suggesting a higher intrinsic repair capacity.

**Figure 6:**
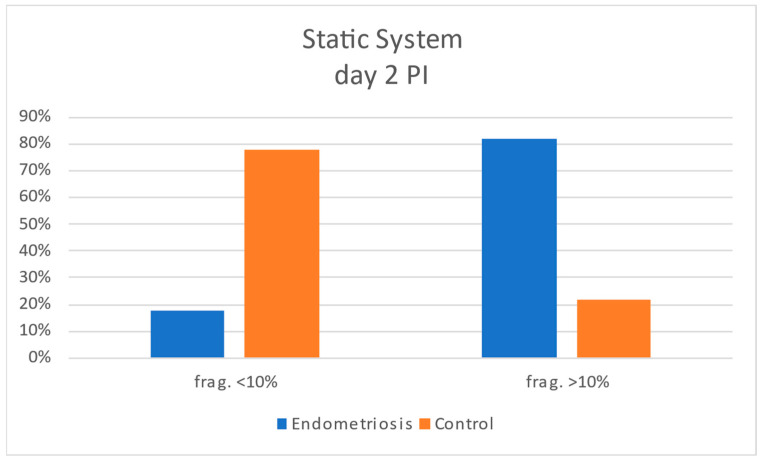
Histogram comparing the degrees of embryonic fragmentation between case and control groups on day 2 post-insemination in the static study.

**Figure 7:**
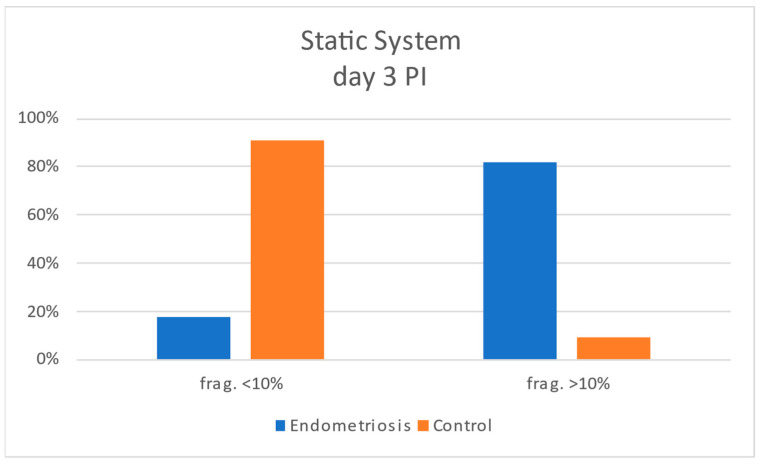
Histogram comparing the degrees of embryonic fragmentation between case and control groups on day 3 post-insemination in the static study.

Static assessments on days 2 and 3 post-insemination confirmed this pattern: embryos from the endometriosis group displayed progressive worsening of fragmentation, while controls tended to improve or remain stable (Figure 6 and Figure 7). Morphokinetic staging also showed comparable developmental distributions between groups, with minor differences in proportions of 2C, 4C, 8C, and 10C stages at each time point, but no significant shift in overall cleavage dynamics.

Regarding clinical outcomes, in the dynamic group, 2 out of 9 endometriosis patients tested positive for β-hCG (28.6%), with one miscarriage (14.3%). In the control group, 7 of 9 patients had a positive β-hCG (78%), including 2 miscarriages (22%). In the static group, 3 of 9 endometriosis patients tested β-hCG positive (33%), with one miscarriage (11%), while in the control group, 4 of 9 patients had a positive β-hCG (44.4%) and no miscarriages. Overall, the biochemical pregnancy rate was lower in endometriosis patients (5/16, 31.3%) than in controls (11/18, 61.1%), and the miscarriage rate among those who conceived was higher in the endometriosis group (40% vs. 18%).

## 4. Discussion

### 4.1. Main Results

This two-phase study investigated the inflammatory and oxidative microenvironment of the ovarian follicle in women with endometriosis undergoing assisted reproductive technology (ART), integrating systemic, local, and cellular analyses. The main study found that women with endometriosis displayed a significantly more pro-inflammatory cytokine profile in both plasma and follicular fluid (FF), characterized by elevated IL-6 and IL-8, and reduced IL-10 levels. These findings are consistent with previous evidence that endometriosis is associated with systemic and local immune dysregulation [2,12,13]. According to our data, recent findings reported increased levels of IL-6, IL-8 and NF-kB in FF of patients with endometriosis undergoing IVF [14].

The clinical data revealed non-significant trends toward lower oocyte maturity, fertilization rates, and blastocyst formation in the endometriosis group, aligning with earlier findings of impaired ART outcomes in this population [6,9].

The sub-study provides novel insights into the impact of endometriosis on early embryo development by comparing static and dynamic embryo evaluation methods. Consistent with previous reports, women with endometriosis had significantly fewer retrieved oocytes, mature oocytes, and developmentally competent embryos compared to controls, despite undergoing similar stimulation protocols [6,9]. This reduction in embryo yield may reflect both compromised oocyte quality and a pro-inflammatory follicular environment, as supported by findings from the main study.

Notably, dynamic time-lapse monitoring revealed a higher incidence of multinucleation and persistent fragmentation in embryos derived from endometriosis patients. These features are well-recognized markers of reduced developmental competence and are often associated with chromosomal abnormalities and impaired implantation potential. A recent study by Llarena et al. demonstrated a significantly lower proportion of good-quality embryos in patients with endometriosis, who also exhibited a reduced number of embryos available for transfer. Specifically, the study showed that embryos derived from patients with endometriosis displayed significant delays in both early and late developmental events. Moreover, the rate of multinucleation was significantly higher in the endometriosis group (50.0% vs. 44.5%, *p* = 0.003) [15,16].

The tendency of embryos from control patients to maintain or reduce fragmentation over time may reflect greater cellular repair capacity, possibly due to a more favorable follicular microenvironment. This observation aligns with prior studies suggesting that oxidative stress and cytokine imbalance in endometriosis can directly impair cytoskeletal integrity and spindle function during oocyte maturation [17].

Although morphokinetic parameters such as timing of cell divisions did not significantly differ between groups, the qualitative differences in fragmentation and multinucleation highlight the limitations of relying solely on timing-based embryo scoring. This reinforces the potential value of integrating morphological and morphokinetic data in embryo selection, particularly in patients with endometriosis. Furthermore, the lower biochemical pregnancy rates and higher miscarriage rates observed in this subgroup may be at least partially attributable to these subtle but critical embryo quality impairments.

The comparison between static and dynamic evaluation systems also suggests that dynamic monitoring may detect developmental abnormalities not evident in traditional morphological assessments, such as delayed fragmentation reduction or transient multinucleation. This underscores its potential utility in improving embryo selection accuracy in populations with known reproductive challenges.

### 4.2. Limitations

Several limitations must be acknowledged. First, the sample size, particularly in the sub-study (*n* = 18), limits the statistical power to detect more subtle associations and may reduce the generalizability of the findings, which is further constrained by the observational monocentric design. Second, embryo-level outcomes were analyzed within each study group; however, embryos derived from the same patient are not statistically independent, and clustered or multilevel models were not applied due to the limited sample size and the consequent risk of overfitting and unstable estimates. Third, the lack of longitudinal reproductive outcomes (e.g., implantation, live birth) restricts clinical extrapolation and limits the ability to fully relate follicular environmental alterations to clinical success. Finally, given the exploratory intent of the study and the number of cytokines, clinical variables, and embryo developmental parameters assessed, no formal correction for multiple comparisons was performed; therefore, *p*-values should be interpreted with caution due to an increased risk of type I error. Accordingly, these results should be considered hypothesis-generating rather than confirmatory and warrant validation in larger, adequately powered prospective studies with predefined primary endpoints and appropriate statistical adjustment.

Second, the observational and monocentric nature of the study may reduce the generalizability of findings. Third, the absence of longitudinal outcome data (e.g., implantation or live birth rates) restricts clinical extrapolation. Finally, although embryo development was analyzed in detail, the study did not include pregnancy or implantation outcomes, which limits the ability to fully correlate follicular environmental alterations with clinical success.

Among the potential confounding factors, maternal age should be highlighted. Oocyte quality and embryonic development are closely associated with the woman’s age. Even small age differences between groups can alter blastomere division timings and the likelihood of aneuploidy. Failure to control for age may erroneously attribute embryonic alterations to endometriosis when, in fact, age is driving the observed changes in TLM parameters.

Additionally, patients with endometriosis often exhibit reduced ovarian reserve, and a lower ovarian reserve may decrease the number of available embryos and favor the selection of lower-quality embryos.

Another important confounder is the male factor: semen quality and the fertilization method (FIVET vs. ICSI) can influence fertilization rates as well as the timing and pattern of embryonic development.

### 4.3. Implications

Previous studies have proposed the use of antioxidant supplementation to counteract the deleterious effects of oxidative stress in patients with endometriosis undergoing ART, with some showing improved embryo development or fertilization outcomes [18,19,20].

## 5. Conclusions

This prospective, monocentric study identifies associations between endometriosis and alterations in follicular and embryonic parameters, potentially mediated by both systemic and local inflammatory environments. Patients with endometriosis displayed a dysregulated cytokine profile, characterized by higher concentrations of pro-inflammatory mediators (including TGF-β1 and NF-κB) and lower levels of the anti-inflammatory cytokine IL-10 in serum and follicular fluid. These inflammatory features were associated with a reduced ovarian response, lower oocyte yield, impaired oocyte maturation, and less favorable fertilization parameters, although causal relationships cannot be inferred from the present data.

The embryo assessment sub-study showed that oocytes obtained from patients with endometriosis were more frequently associated with embryos exhibiting multinucleation and persistent fragmentation, morphokinetic features that have been previously linked to reduced developmental potential. These abnormalities were more consistently identified using time-lapse monitoring compared with conventional static morphological assessment, suggesting that dynamic embryo evaluation may provide additional descriptive information on early developmental patterns in this clinical context.

Overall, the findings are consistent with the hypothesis that the chronic inflammatory and oxidative stress milieu associated with endometriosis correlates with altered oocyte quality and early embryonic morphodynamics. While advanced embryo assessment technologies and peri-ovulatory interventions such as antioxidant or immunomodulatory approaches warrant further investigation, their clinical impact on ART outcomes cannot be established based on the current study. Larger, multicenter prospective studies incorporating robust reproductive endpoints are required to clarify these associations and to determine their potential relevance for the management of endometriosis-associated infertility.

## Figures and Tables

**Table 1:** Baseline characteristics of the study populations.

**Main Study**	**Control (*n* = 18)**	**Endometriosis (*n* = 29)**	***p*** **-Value**
Age (years)	35.20 ± 3.30	36.86 ± 3.68	ns
BMI (kg/m^2^)	22.84 ± 4.08	22.15 ± 3.72	ns
FSH (UI/mL)	7.64 ± 2.02	9.47 ± 3.82	ns
LH (mlU/mL)	5.95 ± 2.91	5.56 ± 2.60	ns
Estradiol (pg/mL)	44.14 ± 22.44	50.19 ± 18.85	ns
AMH (ng/mL)	2.91 ± 0.29	2.29 ± 0.44	0.009
AFC	14.22 ± 0.93	7.76 ± 0.54	≤0.001
Total gonadotropin dose (IU)	2706 ± 907.5	3119 ± 623.2	ns
Days of stimulation	9.56 ± 1.92	9.03 ± 1.86	ns
Gonadotropin/day ratio	285.4 ± 10.9	350.1 ± 18.9	0.003
Follicles ≥ 16mm at trigger	6.22 ± 4.01	3.59 ± 1.82	0.004
**Sub-study**	**Control (*n* = 18)**	**Endometriosis (*n* = 18)**	***p*** **-value**
Age (years)	34.5 ± 2.1	36.6 ± 2.9	0.11
BMI (kg/m^2^)	22.3 ± 2.0	23.1 ± 2.3	0.52
FSH (UI/ml)	7.9 ± 1.2	8.6 ± 1.5	0.26
LH (mlU/mL)	5.2 ± 1.3	5.3 ± 1.6	0.88
Estradiol (pg/mL)	54.6 ± 11.5	50.1 ± 13.9	0.47
AMH (ng/mL)	2.8 ± 0.6	2.2 ± 0.7	0.06
AFC	12.9 ± 3.1	7.8 ± 2.4	0.05
Total gonadotropin dose (IU)	2250 ± 340	2400 ± 360	0.39
Days of stimulation	9.9 ± 0.8	10.3 ± 1.0	0.43
Gonadotropin/day ratio	227 ± 32	233 ± 40	0.72
Follicles ≥ 16mm at trigger	6.1 ± 2.1	4.0 ± 1.5	0.07

“ns” not significat

**Table 2:** Oocyte and embryo quality assessment in endometriosis and control groups.

	Control (*n* = 18)	Endometriosis (*n* = 28)	*p*-Value
Oocyte			
Oocytes retrieved	6.00 ± 0.62	3.86 ± 0.36	0.001
MII oocytes	4.94 ± 0.50	3.36 ± 0.32	0.004
Oocyte anomalies	0.06 ± 0.24	0.18 ± 0.39	ns
Fertilized oocytes (2PN)	4.11 ± 0.58	2.89 ± 0.35	0.031
Embryo			
Grade I embryos	57%	42%	ns
Grade II embryos	25%	27%	ns
Grade III embryos	18%	30%	ns
Blastocyst formation rate	32%	21%	ns
β-hCG positive rate	68%	40%	0.043

“ns” not significant; “2PN” 2 Pro Nuclear.

**Table 3:** Serum and follicular biomarkers in endometriosis and control groups.

Biomarker	Control (*n* = 12)	Endometriosis (*n* = 14)	*p*-Value
Serum markers			
TGF-β1 (pg/5 μg protein)	1542 ± 738	1796 ± 699	0.04
HIF-1α (ng/100 μg protein)	4.48 ± 2.78	11.17 ± 14.67	ns
NF-κB (OD, RU)	5827 ± 2510	8984 ± 5867	0.03
IL-10 (pg/5 μg protein)	33.4 ± 29.4	18.7 ± 11.5	0.04
FF markers			
TGF-β1 (pg/5 μg protein)	1769 ± 1312	1121 ± 506	ns
HIF-1α (ng/100 μg protein)	2.29 ± 2.66	2.12 ± 0.84	ns
NF-κB (OD, RU)	15,573 ± 7887	22,409 ± 47,231	0.04

“ns” not significant.

**Table 4:** Comparison between the endometriosis group and the control group regarding the mean timing of the early stages of embryonic development.

Dynamic System	Endometriosis Group Mean ± SD (hours)	Control Group Mean ± SD (hours)
Number of patients	9	9
Mean PI–syngamy time		
All 2PN embryos	28 ± 3.7	27 ± 3.7
Developmentally competent embryos only	27.5 ± 3.6	27 ± 3.6
Mean PI–first cell division time		
All 2PN embryos	31 ± 4.8	30 ± 3.8
Developmentally competent embryos only	30 ± 3.7	30 ± 3.5
Duration of the 2-cell stage		
All 2PN embryos	13 ± 2.6	12.5 ± 3.3
Developmentally competent embryos only	12 ± 2	12.5 ± 3.6
Mean PI–second cell division time		
All 2PN embryos	43.5 ± 5.3	43 ± 6.8
Developmentally competent embryos only	42.5 ± 4.7	42 ± 5.4

## Data Availability

The original contributions presented in this study are included in the article/supplementary material. Further inquiries can be directed to the corresponding author.

## Supplementary Materials

The following supporting information can be downloaded at https://www.mdpi.com/article/10.3390/jcm15051723/s1. File S1. Additional methodological details of the main study. File S2. Additional methodological details of the sub-study. Table S1. The Istanbul consensus update: a revised ESHRE/ALPHA consensus on oocyte and embryo static and dynamic morphological assessment. Table S2. Consensus scoring system for blastocysts. The Istanbul consensus update: a revised ESHRE/ALPHA consensus on oocyte and embryo static and dynamic morphological assessment.
