# Supplementary material for: Endometriosis-Related Impairment in Assisted Reproductive Technologies: Inflammatory Profiles, Oocyte Competence, and Embryo Development
Source: J Clin Med. 2026 Feb 25;15(5):1723. doi: 10.3390/jcm15051723 (PMC12986511; doi:10.3390/jcm15051723)
Supplement: Supplementary file 1 [file jcm-15-01723-s001.zip › jcm-4048964-supplementary.pdf]

File S1. Additional methodological details of the main study

The initial dose and type of gonadotropin used during controlled ovarian stimulation were individualized based on age, BMI, and ovarian reserve markers, including AMH and antral follicle count (AFC), both assessed on day 3 of the menstrual cycle. AFC was evaluated by a transvaginal ultrasound performed by an experienced operator.

Ovarian response was monitored by serial transvaginal ultrasound and serum estradiol and progesterone measurements starting on the day of stimulation initiation (day 2–3), after 5 days of stimulation, and subsequently every 48 hours until criteria for ovulation trigger were met. A GnRH antagonist (0.25 mg/day) was added when at least one follicle reached a mean diameter of ≥14 mm. Final oocyte maturation was triggered when at least one follicle measured ≥18 mm, using either recombinant hCG (250 μg) or GnRH agonist (triptorelin 0.2 mg), depending on the risk of ovarian hyperstimulation syndrome (OHSS) or need to postpone embryo transfer.

Follicular fluid was collected from a "pure" follicle prior to any washing with culture medium, to avoid dilution of the inflammatory markers. Cumulus cells were retrieved during partial denudation of the oocytes prior to fertilization. Samples of serum, follicular fluid, and cumulus cells were centrifuged at 30,000 rpm for 15 minutes to separate serum from the cellular fraction. Supernatants were aliquoted and stored at –80°C until analysis.

Protein concentration in serum and follicular fluid was measured using the Bradford assay, and levels of inflammatory markers were normalized to total protein. NF-κB levels were quantified using Western blotting. Briefly, 30 μg of protein from serum or follicular fluid were mixed with Laemmli buffer, separated by SDS-PAGE (7.5% polyacrylamide), and transferred to PVDF membranes. Membranes were incubated overnight at 4°C with anti-p65 NF-κB primary antibody. β-actin was used as a loading control. Detection was performed using a suitable secondary

antibody and enhanced chemiluminescence (ECL). Band intensity was quantified by densitometric analysis using ImageJ software.

HIF-1α was measured by a specific ELISA kit (RAB1057-1KT, Sigma-Aldrich, Italy). IL-10 and TGF-β1 concentrations were measured using ELISA kits from Cloud-Clone Corp. (Katy, TX, USA), following the manufacturer's protocols. For IL-10 and TGF-β1, follicular fluid and serum samples were diluted 1:10 before measurement. All absorbance values were read at 450 nm.

Embryo grading was performed according to established morphological criteria, and any visible oocyte abnormalities were recorded. The type of embryo transfer (fresh vs. frozen-thawed) was based on progesterone levels on the day of trigger and clinical judgment related to OHSS risk.

File S2. Additional methodological details of the sub-study

Embryo evaluation under the static protocol was performed using conventional inverted microscopy at defined time points (day 1, 2, and 3 post-insemination). Embryos were cultured in SAGE 1-Step HSA medium, without medium renewal, until the blastocyst stage. Morphological scoring followed Istanbul Consensus[1] criteria and included grading of cleavage speed, symmetry, blastomere number, and degree of fragmentation. Embryos were removed from the incubator daily for observation, and exposure to ambient conditions was minimized but not eliminated.

The dynamic assessment was conducted using the GERI time-lapse incubator (Genea Biomedx), equipped with independent culture chambers, each with high-resolution cameras capturing images every 5–10 minutes. Each embryo was cultured individually in a micro-well of a GERI dish using GERI Medium, supplemented with human serum albumin and gentamicin. This system enabled uninterrupted monitoring under stable environmental conditions (37°C, 5% O_2, 5% CO_2). Image sequences were analyzed to extract developmental timings (pronuclear fading, first and second mitosis, 2-cell stage duration) and qualitative traits such as multinucleation and fragmentation at each cleavage stage.

In the field of Assisted Reproductive Technology (ART), embryo assessment and laboratory protocols have integrated advanced automation and non-invasive diagnostics. Below is the scientific translation and technical detailing of embryo assessment, laboratory assays, and quality control systems.

Embryo Assessment Methodologies

The evaluation of embryonic viability has transitioned from static observations to dynamic, data-driven modeling. Morphokinetic Profiling (Time-Lapse Technology): Beyond traditional snapshots, laboratories utilize continuous monitoring systems (e.g., EmbryoScope). This allows for

the assessment of cleavage kinetics—the precise timing of cell divisions, which serves as a predictive marker for blastocyst formation and aneuploidy (Table S1).

Traditional Morphology (Gardner Criteria): This remains the clinical baseline, grading blastocysts based on: Expansion degree (Stages 1–6), ICM quality (cellular compaction and size), and TE quality (epithelial integrity and cell number).

Table S1 The Istanbul consensus update: a revised ESHRE/ALPHA consensus on oocyte and embryo static and dynamic morphological assessment [10, 11]

Feature	Top ranking	Intermediate ranking	Low ranking
Number of cells	4 cells on Day 2 or 8 cells on Day 3	>4 cells on Day 2 or >8 cells on Day 3	<4 cells on Day 2 or <8 cells on Day 3
Early cleavage	Early cleavage	No early cleavage	
Cell size	Cell stage-specific	Not cell stage specific	
Fragmentation	None or minimal fragmentation (<10%)	10–25% fragmentation	>25% fragmentation
Multinucleation	No multinucleation at any cell stage	No multinucleation at 4 cell stage	Multinucleated at 4-cell stage
Abnormal cleavage	-	-	Direct cleavage DC2 (2- to 5-cell)
Compaction	Compaction from ≥8-cell stage	No compaction	Compaction before 8-cell stage
RECOMMENDATION	• De-prioritize Day 2/3 embryos with abnormal cleavage: direct cleavage DC1 (1- to 3-cell), irregular chaotic division or reverse cleavage, for transfer.<br>• Extend culture of embryos with abnormal cleavage to blastocyst stage.		

Table S2 Consensus scoring system for blastocysts. The Istanbul consensus update: a revised ESHRE/ALPHA consensus on oocyte and embryo static and dynamic morphological assessment [10, 11]

	Stage	Description
**Stage of expansion**	1	Early blastocyst: blastocoel less than half of the volume of the embryo.
	2	Blastocyst: blastocoel that is half of or greater than half of the volume of the embryo
	3	Full blastocyst: blastocoel completely fills the embryo.
	4	Expanded blastocyst: blastocoel larger than that of the early embryo, with a clearly thinning zona

	5	Hatching blastocyst: trophectoderm starting to herniate through the zona.
	6	Hatched blastocyst: blastocyst has completely escaped from the zona
	Grade	Description
ICM	1	Prominent, easily discernible, with many cells that are compacted and tightly adhered together.
	2	Easily discernible, with several cells that are loosely grouped together
	3	Very few cells visible.
	4	No visible cells or presence of degenerating cells.
	Grade	Description
TE	1	Many cells forming a cohesive epithelium.
	2	Moderate number of cells forming a loose epithelium.
	3	Few and larger cells with poor epithelial formation.
	4	Sparse or degenerating cells surrounding the ICM

Laboratory Assay Procedures

Laboratory workflows are designed to maintain homeostasis and maximize developmental competence.

Oocyte Denudation and Insemination: Enzymatic and mechanical removal of cumulus cells followed by either conventional IVF or Intracytoplasmic Sperm Injection (ICSI), often guided by Spindle Imaging to avoid meiotic spindle damage.

Culture Systems: The use of "Single-Step" or "Sequential" media under oil overlay to prevent evaporation and osmolarity shifts. Culture is typically conducted in tri-gas incubators.

Quality Control (QC) and Quality Assurance (QA)

Stringent QC protocols are mandated to ensure the stability of the microenvironment.

Environmental Monitoring:

VOC Filtration: High-efficiency molecular filters to eliminate Volatile Organic Compounds (VOCs), which are potent embryotoxins.

opH Validation: Regular measurement of the extracellular pH of the culture media using specialized micro-electrodes.

Biological Assays for Consumables:

Mouse Embryo Assay (MEA): A bioassay where 1-cell or 2-cell mouse embryos are cultured to the expanded blastocyst stage to screen for sub-lethal toxicity in plastics and media.

KPI Benchmark Analysis: Continuous tracking of Key Performance Indicators, such as the Total Fertilization Rate, Blastocyst Conversion Rate, and Transfer-to-Pregnancy Ratio, as per ESHRE (European Society of Human Reproduction and Embryology) and ASRM guidelines[2].

Semen preparation was standardized using discontinuous density gradient centrifugation (40%/80% silica colloid layers), isolating highly motile sperm for insemination. Fertilization check was performed at 16–20 hours post-insemination, and only normally fertilized (2PN) oocytes were included in subsequent analysis.

Fragmentation was assessed morphologically in the static group on day 2 and day 3, and in the dynamic group immediately after each cell division. Embryos were also categorized by developmental stage (e.g., 2C, 4C, 8C, 10C) at 44 ± 2h and 69 ± 2h post-insemination. Pregnancy outcomes were evaluated via β-hCG measurement 14 days after embryo transfer, and follow-up on miscarriage or ongoing pregnancy status was recorded.
